# Inflammasome‐related gene signatures as prognostic biomarkers in osteosarcoma

**DOI:** 10.1111/jcmm.18286

**Published:** 2024-05-14

**Authors:** Nan Zhang, Ying Han, Hangkai Cao, Qingxin Wang

**Affiliations:** ^1^ Department of Medical Oncology China Coast Guard Hospital of the People's Armed Police Force Zhejiang China; ^2^ Department of Orthopaedic Surgery, Sir Run Run Shaw Hospital Zhejiang University School of Medicine Zhejiang China; ^3^ Key Laboratory of Musculoskeletal System Degeneration and Regeneration Translational Research of Zhejiang Province Zhejiang China; ^4^ Department of Orthopaedic Surgery China Coast Guard Hospital of the People's Armed Police Zhejiang China

**Keywords:** gene signatures, immune profiling, inflammasomes, osteosarcoma, prognostic biomarkers

## Abstract

Osteosarcoma, the primary bone cancer in adolescents and young adults, is notorious for its aggressive growth and metastatic potential. Our study delved into the prognostic impact of inflammasome‐related gene signatures in osteosarcoma patients, employing comprehensive genetic profiling to uncover signatures linked with patient outcomes. We identified three patient subgroups through consensus clustering, with one showing worse survival rates correlated with high FGFR3 and RARB expressions. Immune profiling revealed significant immune cell infiltration differences among these subgroups, affecting survival. Utilising advanced machine learning, including StepCox and gradient boosting machine algorithms, we developed a prognostic model with a notable c‐index of 0.706, highlighting CD36 and MYD88 as key genes. Higher inflammasome risk scores from our model were associated with poorer survival, corroborated across datasets. In vitro experiments validated CD36 and MYD88's roles in promoting osteosarcoma cell proliferation, invasion and migration, emphasising their therapeutic potential. This research offers new insights into inflammasomes' role in osteosarcoma, introducing novel biomarkers for risk assessment and potential therapeutic targets. Our findings suggest a pathway towards personalised treatment strategies, potentially improving patient outcomes in osteosarcoma.

## 1 INTRODUCTION

Osteosarcoma, the most prevalent malignant bone tumour in paediatric populations, presents a stark embodiment of cancer's ruthless disregard for life's potential.^1^, ^2^ This aggressive disease typically strikes in the bloom of youth, interrupting the lives of adolescents as they stand on the cusp of adulthood.^3^, ^4^ Despite remarkable progress in cancer research, the overall survival rate for osteosarcoma has plateaued over the past few decades, especially for those with metastatic disease at diagnosis.^5^, ^6^, ^7^ The stagnation of therapeutic advancements underscores an urgent need for novel approaches in understanding and treating this malignancy.^8^, ^9^

The battle against osteosarcoma is fought on a molecular frontier, where the identification of key genetic and immunologic factors holds the promise of turning the tide in patient outcomes.^10^, ^11^ Recent scientific inquiries have directed a spotlight onto inflammasomes—complexes involved in the innate immune response—as pivotal players in the cancer saga.^11^, ^12^ These inflammasomes, which orchestrate a cascade of inflammatory responses, have emerged as double‐edged swords within the tumour microenvironment.^13^, ^14^ Their role in cancer is as paradoxical as it is central: they can both impede and incite tumour progression, making their study crucial for the development of targeted therapies.^15^

Understanding the dance between osteosarcoma cells and the immune system reveals a landscape rife with potential targets for intervention.^16^ The immune system, traditionally viewed as an army defending against the onslaught of neoplastic transformation, has been found to be co‐opted at times, aiding and abetting tumour growth and spread.^17^, ^18^ Chronic inflammation, a hallmark of immune involvement, has been linked to various stages of tumorigenesis, from initiation to metastasis, presenting a challenging yet critical target for therapeutic development.^19^

The heterogeneity of osteosarcoma at the molecular level presents both a challenge and an opportunity for researchers.^20^, ^21^ The advent of high‐throughput genomic and proteomic technologies has paved the way for the in‐depth characterisation of this heterogeneity, allowing for the subdivision of osteosarcomas into molecularly distinct subtypes.^22^, ^23^ This stratification is not merely academic; it bears direct implications for prognosis and therapeutic strategies, guiding the course of precision medicine—a tailored approach that aligns treatment with individual tumour profiles.^24^, ^25^

In the quest to unravel the complexities of osteosarcoma, this study is strategically designed to explore the molecular underpinnings of the disease, placing a specific emphasis on inflammasome‐related gene signatures. This focus is driven by a dual intent: to deepen the collective understanding of the pathophysiology underlying osteosarcoma and to scrutinise the prognostic significance of these genes. Uncovering this knowledge is not merely academic; it holds the tangible potential to identify patients predisposed to adverse outcomes, thereby refining therapeutic approaches and potentially revealing novel targets for treatment. The impetus behind this research stems from an essential lacuna in our grasp of how osteosarcoma interacts with the immune system. Although inflammation's role in oncogenesis is well‐established, the distinct impact of inflammasome‐related genes on the evolution of osteosarcoma is not well characterised. This investigation seeks to elucidate the influence of these genes on tumour dynamics and patient survival, contributing to a broader scientific quest to demystify cancer. This work, built upon the extensive research conducted over previous decades, endeavours to push the boundaries of current cancer knowledge. By delving into the confluence of genetics, immunology, and oncology, the study is poised to transform the current paradigm of osteosarcoma from one shrouded in uncertainty to one illuminated by insight and hope, offering new avenues for improved patient care and outcomes.

## 2 METHODS

### 2.1 Data collection and preprocessing

In the data collection phase, we meticulously gathered and processed data from two distinct datasets and a specific gene set. Our primary dataset, the training set, consisted of 93 osteosarcoma patients from the TARGET‐OS cohort. This dataset included both matched survival data and comprehensive clinical information for each patient. The gene expression data for this cohort were processed using the DESeq2 package, which facilitated normalization and variance‐stabilising transformation (VST) of the raw count data.^26^ Additionally, we utilised a validation set, GSE39055, comprising 37 osteosarcoma patients, each with matched survival data. The expression matrix for this validation set was standardised and normalized using the limma package to ensure data consistency and reliability.^27^ To enrich our analysis, we also incorporated a specific gene set focusing on inflammasome‐related genes. This gene set was acquired from the GSEA official website and included genes from three pathways related to inflammasome activity. After deduplicating these genes, we compiled a list of 26 unique inflammasome‐related genes for further investigation in our study, aiming to provide a comprehensive understanding of the genetic underpinnings of osteosarcoma.

### 2.2 Identification of inflammasome‐related genes and clustering for survival analysis in osteosarcoma

In our study, we aimed to identify inflammasome‐related genes from the TARGET training set, successfully recognising 24 out of 26 genes. To understand the expression patterns of these genes, we performed consensus clustering based on their standardised expression levels. For this task, the ConsensusClusterPlus package was employed to determine the optimal number of clusters. Survival analysis was conducted to investigate the statistical significance of the survival differences between the identified subgroups. This was achieved using the survival package for survival analysis and the survminer package to plot the survival curves, which facilitated the visualisation of survival discrepancies among the subgroups.^28^ To explore the expression of potential regulatory factors and transcription factors related to cancer chromatin remodelling within the subgroups, we referred to the literature to collect a relevant set of genes. Enrichment scores for these gene sets were computed using the GSVA package, which allowed us to assess the level of activity of these genes in the context of chromatin remodelling. Additionally, we evaluated the GSVA scores for three gene sets related to treatment response, which were also gathered from the literature.^29^ The ComplexHeatmap package was utilised to visualise these scores in a heatmap format, providing insights into the potential treatment responsiveness and malignancy of the subgroups.

### 2.3 Immune abundance scoring and inflammasome‐related gene selection in osteosarcoma

For the analysis of immune cell abundance across different subgroups of osteosarcoma, we used the Cibersortx online platform (https://cibersortx.stanford.edu/) to calculate the relative abundance of 22 types of immune cells. The immune abundance scores were visualised using box plots created with the ggpubr package, and the differences in immune cell types among the subgroups were further detailed using alluvial diagrams created with the ggalluvial package. To assess the myeloid‐derived suppressor cell (MDSC) abundance as part of the tumour immune dysfunction and exclusion (TIDE) evaluation, we utilised the TIDE online tool (http://tide.dfci.harvard.edu). The MDSC scores were visualized using box plots generated with the ggpubr package, providing insights into the immune contexture of the subgroups. Differences in clinical information composition among the subgroups were visualized using stacked bar plots, created with the ggplot2 package. This analysis included the proportions of metastasis and patient survival outcomes across the subgroups.

### 2.4 Machine learning‐based selection of inflammasome‐related genes for osteosarcoma prognosis

For the task of selecting inflammasome‐related genes associated with overall survival in osteosarcoma, we employed a comprehensive machine learning approach, integrating 10 distinct methods into 101 different model‐building strategies. This involved the use of several R packages, each tailored to survival analysis in unique ways. The LASSO method in the glmnet package used least squares with a penalty term to shrink coefficients of less crucial variables to zero, setting its alpha value to one. The Elastic Net, also within glmnet, balanced the LASSO and Ridge penalties by adjusting the alpha coefficient between 0 and 1. The Stepcox method from the survival package employed a step function combined with Cox regression to select models based on the smallest Akaike Information Criterion (AIC). SurvivalSVM, implemented via the survivalsvm package, conducted survival analysis through support vector machines, adding a higher‐dimensional plane for data segmentation. CoxBoost integrated Cox regression models with boosting algorithms to enhance predictive performance while retaining interpretability. The SuperPC method combined principal component analysis with supervised learning to yield a supervised principal component analysis approach. Ridge regression, akin to LASSO but setting alpha to zero, was used for variable selection in glmnet. PlsRcox in the plsRcox package fitted Cox models in high‐dimensional settings using partial least squares regression techniques. Random Survival Forest (RSF) from the randomForestSRC package predicted survival outcomes using random forest models. Finally, Gradient Boosting Machines (GBM) in the gbm package serially generated multiple weak learners, each fitting the negative gradient of the loss function of the preceding cumulative model. Employing various machine learning techniques yields substantial benefits in accurately pinpointing and validating essential genes pivotal for prognostic assessments across different diseases.^30^, ^31^

### 2.5 Immune cell abundance and immune activity analysis in osteosarcoma based on Inflammasome Score

To evaluate the immune cell abundance in the training set of osteosarcoma patients, we utilised a deconvolution approach with seven distinct computational methods for immune cell quantification. This comprehensive analysis was facilitated by the IOBR package, which includes a suite of algorithms such as Cibersort, EPIC, MCPCounter, xCell, Estimate, Timer, QUANTISEQ and IPS. Each of these algorithms specialises in assessing the relative enrichment scores of different immune cell types through deconvolution, providing a thorough perspective on the immune landscape within the tumour microenvironment. In addition to immune cell profiling, we performed single‐sample Gene Set Enrichment Analysis (ssGSEA) on 255 classic tumour‐related gene sets, which are listed in the ‘signature_collection_citation.txt’ file from the original data.^32^ This ssGSEA scoring was followed by a univariate Cox regression analysis to calculate hazard ratios (HR) and *p*‐values for each gene set. The results, particularly the top 20 pathways, were visualized using forest plots to elucidate the protective or risk nature of these pathways in relation to osteosarcoma. The inflammasome score (ISS) is a composite metric that quantifies the activity of the inflammasome complex within a biological sample by aggregating the expression levels of its constituent genes and proteins. Furthermore, to understand the immune characteristics associated with high and low ISS score groups, we evaluated the expression of various immune activity molecules, including inflammatory chemokines, TNF family molecules, and HLA family molecules. The introdataviz and ggpubr packages were employed to create split violin plots, which effectively illustrate the differences in expression levels of these immune molecules between the high and low ISS score groups. This approach allows us to infer the chemotactic activity, immune‐inflammatory response, and antigen‐presenting capabilities within these groups.

### 2.6 Pathway evaluation and correlation analysis in osteosarcoma based on ISS scores

For the comprehensive pathway evaluation of osteosarcoma based on ISS, we conducted GSEA focusing on three key gene sets: HALLMARK, GOBP and KEGG, using the IOBR package.^33^ This analysis aimed to discern the functional activities and biological processes associated with high and low ISS groups. In the HALLMARK gene set analysis, we investigated the enrichment of pathways like epithelial–mesenchymal transition (EMT) and interferon response, particularly in the low ISS score group. The GOBP gene set was analysed to identify significant biological processes such as adaptive immune response and lymphocyte activation regulation. For the KEGG pathways, our focus was on cellular processes like cytokine–cytokine receptor interaction. Additionally, we performed a correlation analysis between the ISS score and three classical tumour‐related processes: angiogenesis, cell cycle and EMT. Gene sets for these processes were extracted from specific literature sources, and the correlations with ISS scores were visualized using coloured scatter plots created with ggplot2 and ggpubr packages. The angiogenesis gene set,^34^ the cell cycle gene set,^35^ and the EMT gene set^36^ were each analysed for their correlation with the ISS score. These comprehensive analyses provided a nuanced understanding of the biological characteristics and potential driving mechanisms behind different ISS score groups in osteosarcoma, contributing to a deeper understanding of the disease's pathology.

### 2.7 Pan‐cancer analysis of prognostic genes CD36 and MYD88

In the context of cancer biology, CD36 and MYD88 stand out due to their pivotal roles in modulating tumour progression and immune responses. CD36, a transmembrane glycoprotein, is widely recognised for its function in fatty acid metabolism and has been increasingly implicated in the facilitation of tumour growth and metastasis. Its expression in various cancer types has been linked to enhanced fatty acid uptake, promoting an energy‐rich environment conducive to rapid tumour cell proliferation. Moreover, CD36 has been observed to contribute to the tumour microenvironment's remodelling, aiding in tumour invasion and metastasis. On the other hand, MYD88 is an essential adaptor protein in the toll‐like receptor (TLR) and IL‐1 receptor signalling pathways, playing a critical role in innate immune responses. In the context of cancer, MYD88 signalling has been associated with inflammatory responses that can either suppress or promote tumour development, depending on the tumour type and the surrounding microenvironment. Its role in mediating pro‐inflammatory cytokine production makes it a key player in the tumour‐promoting inflammation and in shaping the immune landscape within the tumour microenvironment. Understanding the multifaceted roles and mechanisms of CD36 and MYD88 in cancers is crucial for elucidating their potential as therapeutic targets or prognostic markers. Their involvement in both metabolic regulation and immune response modulation positions them as significant contributors to the complex interplay between tumour cells and their microenvironment, influencing tumour progression, metastasis and the response to therapy.

In our pan‐cancer study, we focused on the core prognostic genes CD36 and MYD88, utilising the TCGAplot package to perform a broad analysis and visualisation across various cancer types. We assessed the expression levels of these genes in multiple cancers, comparing them to control groups to understand their differential expression. Our investigation extended to the tumour microenvironment, where we analysed the correlation of these genes with stromal, immune and tumour microenvironment scores, using the insights to comprehend their interactions within the tumour context. We further explored their relationship with key immunological factors, including immune checkpoints and tumour mutational burden (TMB), to gauge their potential influence on immune response and relevance in immunotherapy. The analysis also included examining the correlation of CD36 and MYD88 with microsatellite instability (MSI) across different cancers. Additionally, we employed Cibersort to conduct a pan‐cancer immune cell abundance analysis. This approach allowed us to understand the associations of CD36 and MYD88 with various immune cell types and their impact on the immune landscape of cancer. We also analysed the expression of these genes in the context of clinical subtypes across various cancers, exploring differences in gene expression at different stages of cancer progression. The study concluded with a survival analysis assessing the relationship between the expression of CD36 and MYD88 and patient survival in various cancers. We used the TCGAplot package to visualise this relationship and understand the prognostic significance of these genes across a range of cancer types. This comprehensive pan‐cancer analysis aimed to provide a multifaceted view of the roles of CD36 and MYD88, underlining their expression patterns, interactions with the tumour microenvironment and immune system, and their overall clinical and prognostic relevance.

### 2.8 Cell culture and transfection

In this study, the human osteosarcoma cell line U‐2 was utilised for wet lab validation experiments. The U‐2 cell line was procured from the Cell Bank of the Chinese Academy of Sciences. McCoy's 5A medium (Solarbio, China) supplemented with 10% fetal bovine serum (FBS, Solarbio, China) and 1% penicillin–streptomycin solution (BI, Israel) was used for cell culture. Cell culture flasks were maintained at 37°C in a humidified cell culture incubator with 5% CO_2_, ensuring optimal logarithmic growth of the cells. Passaging of the cells was performed every 36 h.

For transfection experiments with the U‐2 cell line, siRNA targeting CD36 and MYD88 was designed and produced by a commercial biotechnology company (GIMA Corporation, China) to achieve gene knockdown. Initially, cells were detached from the culture flasks using trypsin (Thermo Fisher, USA), washed with phosphate buffered saline (PBS, Solarbio, China), and centrifuged to suspend the cells in the culture medium. Subsequently, cells were seeded onto six‐well plates at a concentration of 3 × 104 cells per well and supplemented with culture medium to a total volume of 2 mL per well. Following cell attachment, siRNA was mixed with the transfection reagent FuGENE® SI (Fugene, USA) according to the manufacturer's instructions. After 30 min of incubation at room temperature, the siRNA‐transfection reagent mixture was evenly injected into the wells of the six‐well plate. The culture medium was replaced after 4 h of transfection, and subsequent experiments were conducted 48 h post‐transfection. The sequences of the siRNAs were as follows: si‐CD36: 5′–UGCUUAUCCAGAAGACAAU‐3′; si‐MYD88: 5′–ACCAACUUUGUACCUUGAU‐3′.

### 2.9 Total RNA extraction and RT‐qPCR

To evaluate the efficiency of transfection experiments, RT‐qPCR was performed to detect the expression differences of CD36 and MYD88 between their respective transfection groups and control groups. Forty‐eight hours post‐transfection, the culture medium was removed, and cells were gently washed with PBS. Cells were then detached from the wells of the six‐well plate using trypsin, washed with PBS, and centrifuged at low temperature. The supernatant was discarded, and the cell pellet was retained. Following the manufacturer's protocol, cells were lysed using Trizol (Takara, Japan) to release RNA. After repeated pipetting and incubation on ice for 5 min, chloroform (Beyotime, China) was added to facilitate phase separation, resulting in the isolation of RNA from DNA and proteins. The aqueous phase containing RNA was transferred to a RNase‐free centrifuge tube, and isopropanol (SINOPHARM, China) was added to precipitate RNA. After centrifugation, the supernatant was discarded, and the RNA pellet was washed and dried with 75% ethanol (SINOPHARM, China). After each addition of a liquid, the following steps were performed: thorough mixing by vortexing, centrifugation at 4°C and 12,000 rpm for 15 min, and incubation on ice. Following these steps, all organic solvents were removed, and the centrifuge tube was left on a clean bench for 40 min to obtain a fluffy RNA pellet. The pellet was dissolved in 20 μL of diethyl pyrocarbonate (DEPC) water, and subsequent procedures were carried out according to the instructions of different instruments or reagent kits. The RNA concentration was measured using a Nanodrop2000 instrument (Thermo, USA), and cDNA was synthesised from RNA using the PrimeScript™ RT reagent Kit (Takara, Japan). The cDNA solution was diluted to ensure a CT value >15, and SYBR GreenER Supermix (TaKaRa, Japan) was used for pre‐mixing of cDNA samples before loading onto the machine. Following the parameters provided by the SYBR GreenER Supermix (TaKaRa, Japan) kit, real‐time fluorescent quantitative PCR was performed on the 7500 Real‐Time PCR System (Thermo Fisher, USA). Finally, the relative expression of CD36 and MYD88 was analysed using the 2–ΔΔCt method with β‐actin as an internal reference. The primer sequences were as follows: CD36‐F: 5′‐AGTTTGGTTCCGTACCCTGT‐3′; CD36‐R: 5′‐TGAGGCTGCATCTGTACCAT‐3′; MYD88‐F: 5′‐GGCACCTGTGTCTGGTCTAT‐3′; MYD88‐R: 5′‐TTCTGATGGGCACCTGGAG‐3′.

### 2.10 CCK8 assay

We assessed changes in cell proliferation capacity following knockdown of CD36 and MYD88 using the CCK8 assay. After transfection for 48 h, cells from six‐well plates were evenly transferred to 96‐well plates at a density of 4000 cells per well. To ensure reliability, each group was triplicated. The CCK8 reagent (KeyGEN, China) mixed with complete medium was added to each well, and after cell adherence, the original medium was replaced. The total volume per well was 200 μL. Plates were then incubated under light‐avoiding conditions for 1.5 h, followed by measuring the absorbance at 450 nm. This procedure was repeated at 24, 48, and 72‐h time points.

### 2.11 Transwell assay

U‐2 cell line was used to validate changes in cell proliferation capacity post CD36 and MYD88 knockdown. Matrigel (Corning, USA) was diluted in serum‐free medium at a ratio of 1:8 and added to the chamber (Corning, USA) wells. After 24 h of drying, cells from six‐well plates were detached using trypsin, suspended in serum‐free medium, and seeded into the chambers at a concentration of 30,000 cells per chamber with a volume of 230 μL. The culture medium in the 24‐well plate contained FBS (final volume of 650 μL per well). After 24 h of incubation, the chambers were fixed with 4% paraformaldehyde, stained with 0.1% crystal violet (Solarbio, China), washed, air‐dried and photographed under a microscope.

### 2.12 Wound healing assay

We employed the wound healing assay to validate the changes in migration capacity of OS cells following the knockdown of CD36 and MYD88. Forty‐eight hours post‐transfection, a 200 μL pipette tip was used to uniformly and slowly create a scratch in the center of each well of a six‐well plate. Subsequently, the pipette tip and the culture medium were removed, and floating cells were gently washed away with PBS. After at least two washes, 2 mL of basal culture medium without FBS was added to each well. Subsequently, under a microscope with a 100‐fold magnification, photographs were taken of the scratch at this moment, defined as the 0‐h time point, and the scratch area was estimated. The six‐well plate was then placed in a cell culture incubator for 48 h, followed by capturing images of the scratch area using the same magnification, and recording the scratch area. The migration rate was calculated by subtracting the scratch areas before and after from each other. Each group was repeated three times.

### 2.13 EdU assay

Cells from six‐well plates were digested with trypsin and seeded into 96‐well plates at a density of 4 × 104 cells per well after 48 h of transfection. EdU solution (Beyotime, China) was diluted at a ratio of 1000:1 to prepare EdU culture medium, which was added to each well (100 μL per well). After 2 h of incubation, excess EdU medium was removed, and cells were washed with PBS. Cells were fixed with 4% paraformaldehyde, permeabilized with Triton X‐100 (Dow Chemical Company, USA), stained with Hoechst 33342 (Beyotime, China), and observed under a fluorescence microscope after incubation.

### 2.14 Statistical analysis

All analyses and visualisation were performed in R software (version.4.2.1). A value of *p* < 0.05 was used as the criteria for statistical significance.

## 3 RESULTS

### 3.1 Identification of inflammasome‐related genes and cluster analysis

**FIGURE 1:**
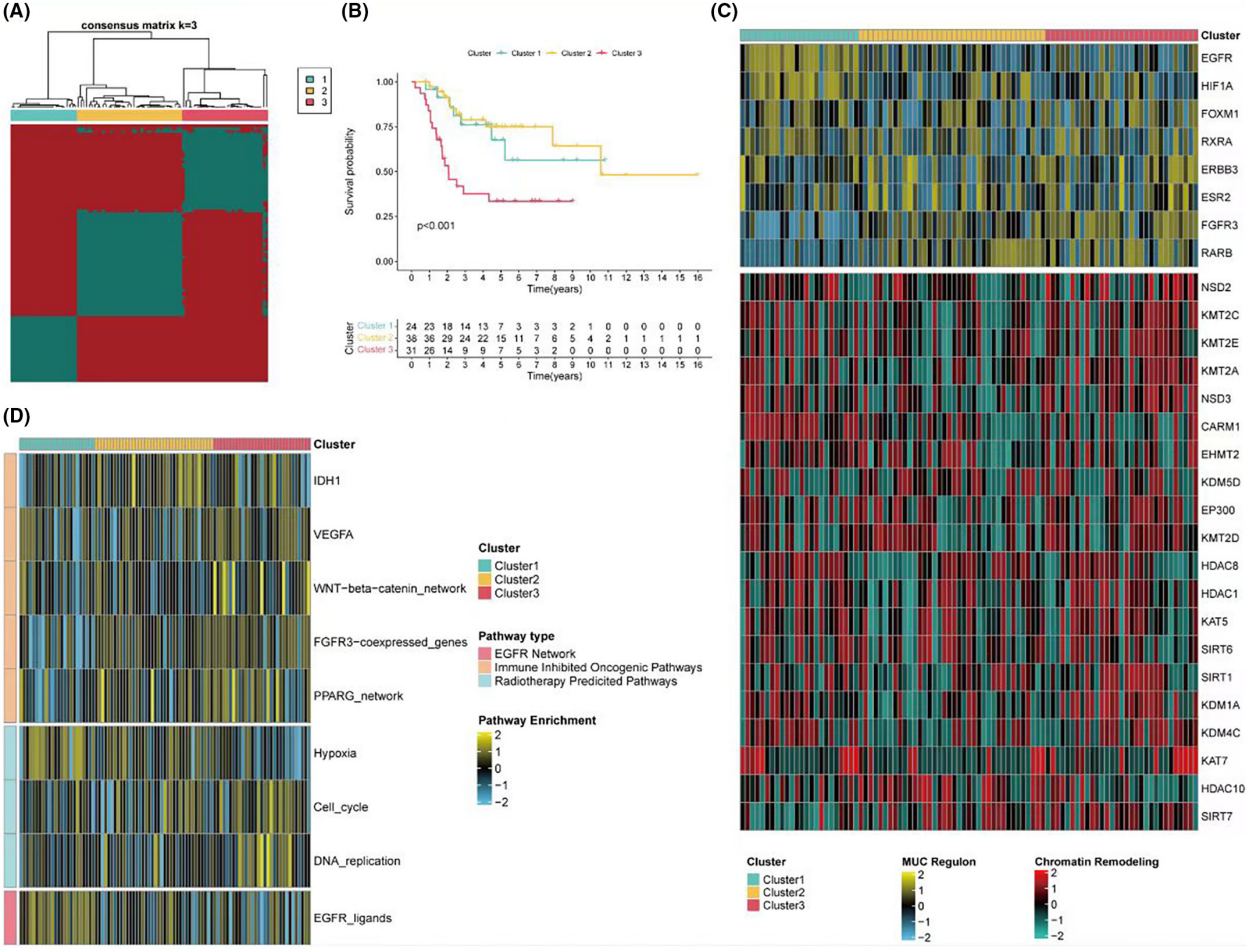
Identification of inflammasome‐related genes and cluster analysis. (A) Consensus matrix heatmap from a *k*‐means clustering algorithm applied to 24 inflammasome‐related genes identifying three distinct patient clusters (*k* = 3) within the TARGET dataset. The optimal number of clusters was determined based on consensus clustering stability. (B) Kaplan–Meier survival curves comparing overall survival between the three identified clusters. Statistical significance was calculated using the log‐rank test, with subgroup 3 demonstrating the worst survival outcome. (C) Heatmap illustrating the expression levels of genes associated with cancer chromatin remodelling across the three patient clusters. High expression levels of FGFR3 and RARB are notable in subgroup 3, whereas EGFR and HIF1A are predominantly expressed in subgroup 1. Subgroup 2 shows low expression across most of the genes. (D) Gene set variation analysis (GSVA) heatmap showing pathway enrichment scores related to treatment response for each cluster. Subgroup 3 is characterised by higher pathway scores suggesting increased treatment responsiveness and potential malignancy.

From the TARGET dataset, 24 inflammasome‐related genes were identified, with two remaining unidentified. Consistency clustering based on the standardised expression levels of these 24 inflammasome‐related genes revealed the best classification results when *k* = 3 (Figure 1A). At this point, there were significant statistical differences in survival among the three subgroups, with subgroup 3 having the worst overall survival rate (Figure 1B). The expression of potential regulatory factors and transcription factors related to cancer chromatin remodelling in the three subgroups was analysed. Subgroup 3 mainly exhibited high expression of FGFR3 and RARB, subgroup 1 primarily showed high expression of EGFR and HIF1A, while subgroup 2 had low expression of most chromatin remodelling genes (Figure 1C). Additionally, the GSVA scores of three gene sets related to treatment response among the subgroups were assessed. The heatmap indicated that subgroup 3 might have higher treatment responsiveness pathway scores, reflecting its malignancy and potential treatability (Figure 1D).

### 3.2 Immune abundance scoring and clinical information analysis

**FIGURE 2:**
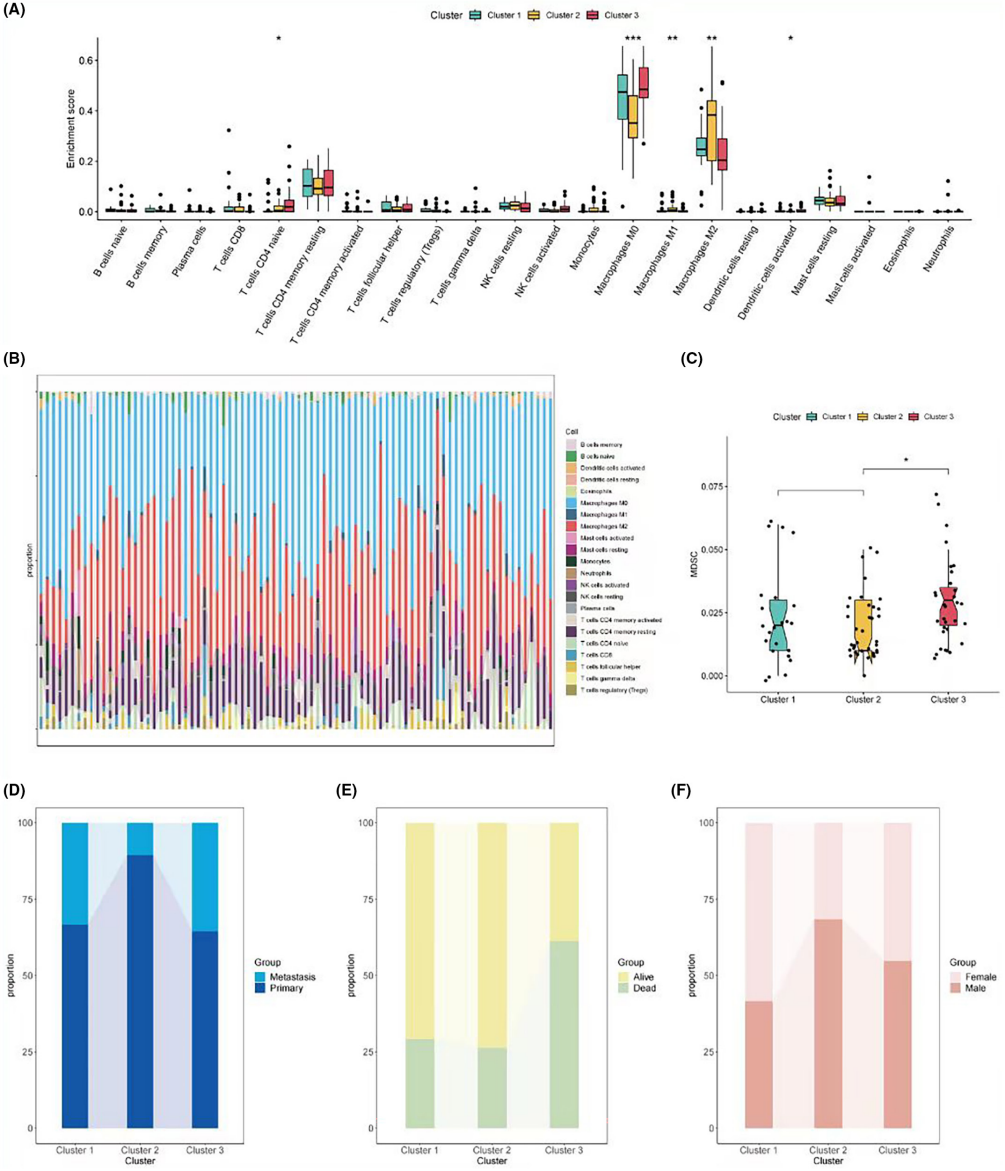
Immune abundance scoring and clinical information analysis. (A) Box plots displaying the abundance scores of CD4 naive T cells, M0 macrophages, M1 macrophages, M2 macrophages and activated dendritic cells among the three patient clusters, derived using the Cibersort algorithm. (B) Bar graph representing the immune cell composition within each cluster, highlighting the significant inter‐cluster differences. (C) Box plot showing the TIDE (tumour immune dysfunction and exclusion) assessment of myeloid‐derived suppressor cell (MDSC) abundance for each cluster, with subgroup 3 exhibiting the highest levels. (D) Bar chart showing the proportion of primary and metastatic cases within each patient cluster. (E) Stacked bar chart representing the survival status (alive or deceased) of patients in each cluster. (F) Stacked bar chart displaying the gender distribution within each patient cluster.

Next, the immune abundance scores of the three subgroups were calculated. The Cibersort algorithm results revealed significant differences among the three groups in CD4 naive T cells, M0, M1, M2, and activated dendritic cells (Figure 2A,B). The TIDE assessment showed the highest MDSC abundance in subgroup 3, which might be associated with its poorer prognosis (Figure 2C). Further analysis of the differences in the composition of various clinical information among the three subgroups showed that subgroup 3 had the highest proportion of metastasis and the highest number of deceased patients, while subgroup 2 had the smallest proportion of metastasis (Figure 2D–F).

### 3.3 Selection of osteosarcoma survival‐related inflammasome genes using machine learning

**FIGURE 3:**
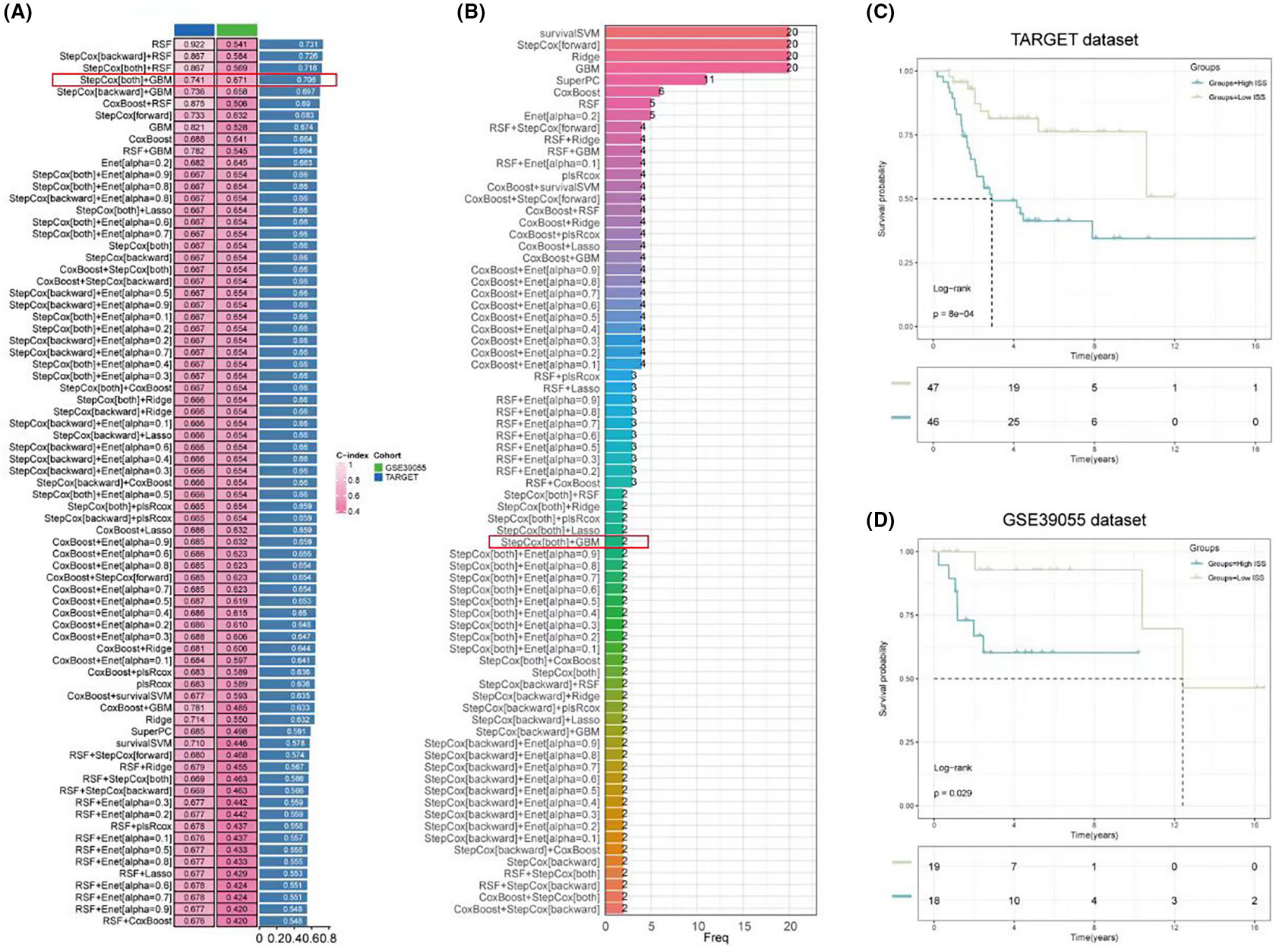
Selection of osteosarcoma survival‐related inflammasome genes using machine learning. (A) Bar chart summarising the c‐index values for 101 different machine learning model combinations applied to the inflammasome‐related genes, highlighting the performance of RSF and the selected StepCox [both] and GBM (gradient boosting machine) combination. (B) Frequency plot showing the selection frequency of different inflammasome‐related genes across the machine learning models, with CD36 and MYD88 emerging as core genes for model construction. (C) Kaplan–Meier survival curves for the TARGET dataset stratified by inflammasome risk score (ISS), indicating a significant association between higher ISS scores and decreased overall survival. (D) Kaplan–Meier survival curves for the GSE39055 dataset stratified by ISS, corroborating the findings from the TARGET dataset regarding the prognostic significance of ISS scores.

To further select inflammasome‐related genes associated with overall survival in osteosarcoma, 101 screening and modelling approaches were employed using a combination of 10 different machine learning methods. The results showed that RSF had the highest predictive performance in model construction. However, due to a large discrepancy in the c‐index values between the training and validation sets, a more stable predictive model was sought. A combination with closer c‐index values and high predictive performance was chosen, namely StepCox [both] for variable selection and GBM for model construction, resulting in an average c‐index of 0.706 (Figure 3A). This screening method ultimately retained only two genes for model construction: CD36 and MYD88 (Figure 3B). Based on the model, an inflammasome risk score (ISS score) was calculated for all samples in the training set. Survival curves indicated that a higher ISS score was significantly associated with poorer overall survival (Figure 3C), a conclusion that was also drawn from the validation set (Figure 3D).

### 3.4 Immune cell profiling and pathway analysis in relation to ISS scores

**FIGURE 4:**
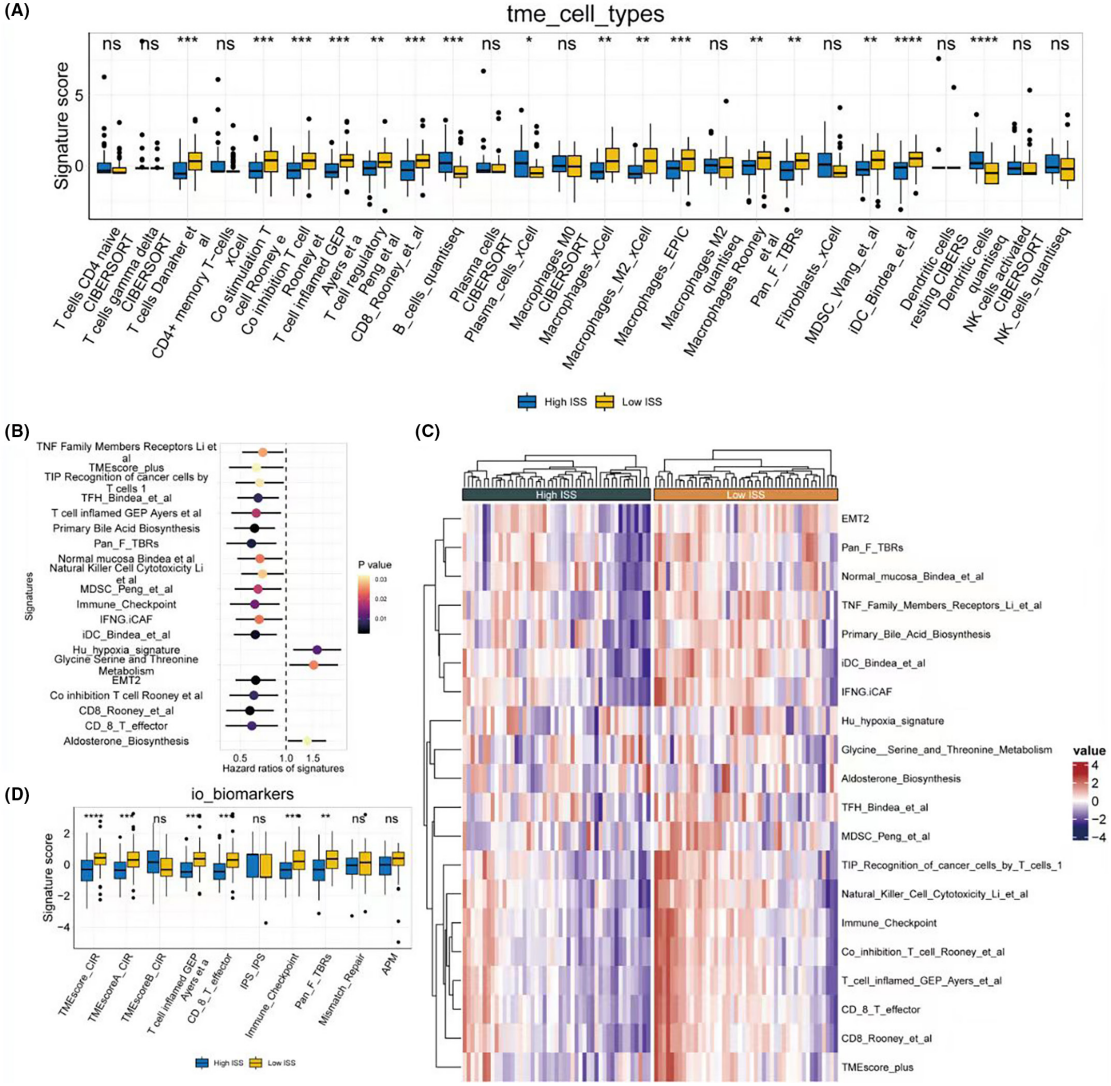
Immune cell profiling and pathway analysis in relation to ISS scores. (A) Bar chart representing the abundance of seven immune cell types in patient samples, with deconvolution analysis showing a higher abundance of T cells, B cells and macrophages associated with lower ISS scores.(B) Forest plot depicting the HRs and *p*‐values from single‐factor Cox regression analyses for the top 20 pathways derived from 255 classic tumour‐related gene sets, with the majority indicating protective pathways (HR <1). (C) Heatmap showing the differential pathway enrichment scores between high and low ISS score groups across the top 20 pathways, suggesting better survival for the low ISS score group with higher enrichment scores.(D) Heatmap of ssGSEA scores for gene sets related to immunotherapy, highlighting the higher scores in the low ISS score group, which suggests a potential benefit from immunotherapy due to higher immune cell abundance.

Based on the expression matrix of the training set, deconvolution using seven types of immune cells was performed. The results showed that a lower ISS score was significantly associated with a higher abundance of immune cells, including T cells, B cells, macrophages, and others (Figure 4A). Using 255 classic gene sets related to tumours (see original data in ‘4‐risk immune’ under ‘signature_collection_citation.txt’), ssGSEA scoring was conducted. Single‐factor Cox regression analysis of HR and *p* values was performed, and the forest plot displayed the top 20 pathways, most of which were protective pathways, that is HR <1 (Figure 4B). Heatmaps illustrated the enrichment scores of the top 20 pathways between high and low ISS score groups, revealing significantly higher enrichment scores in the low ISS score group, suggesting better survival prospects (Figure 4C). Similarly, in the collected gene sets related to immunotherapy, the low ISS score group showed higher scores, indicating that this group not only has a higher abundance of immune cells but may also potentially benefit more from immunotherapy (Figure 4D).

### 3.5 Expression of immune activity molecules in different ISS score groups

**FIGURE 5:**
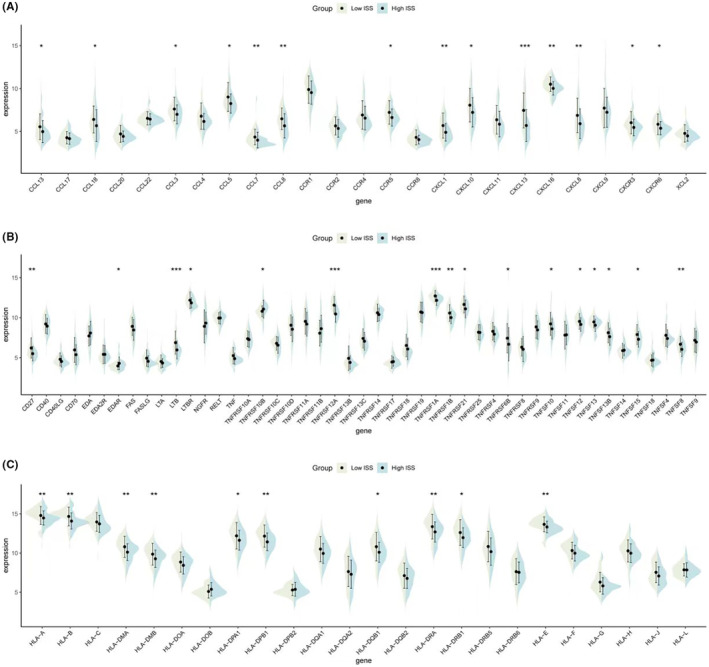
Expression of immune activity molecules in different ISS score groups. (A) Violin plots illustrating the expression levels of inflammatory chemokines across high and low ISS score groups, with the low ISS score group showing higher expression, indicative of enhanced chemotactic activity. (B) Violin plots showing the expression levels of TNF family molecules, with higher expression observed in the low ISS score group, suggesting a more robust immune‐inflammatory response. (C) Violin plots depicting the expression levels of HLA family molecules, with elevated expression in the low ISS score group, which may imply improved antigen‐presenting functions.

Regarding immune activity molecules, the expression of inflammatory chemokines (Figure 5A), TNF family molecules (Figure 5B), and HLA family molecules (Figure 5C) were calculated for groups with high and low ISS scores. Violin plots demonstrated that all three types of molecules were highly expressed in the low ISS score group, suggesting that this group also exhibits better chemotactic activity, immune‐inflammatory responses, and antigen‐presenting functions.

### 3.6 Pathway assessment in relation to ISS scores

**FIGURE 6:**
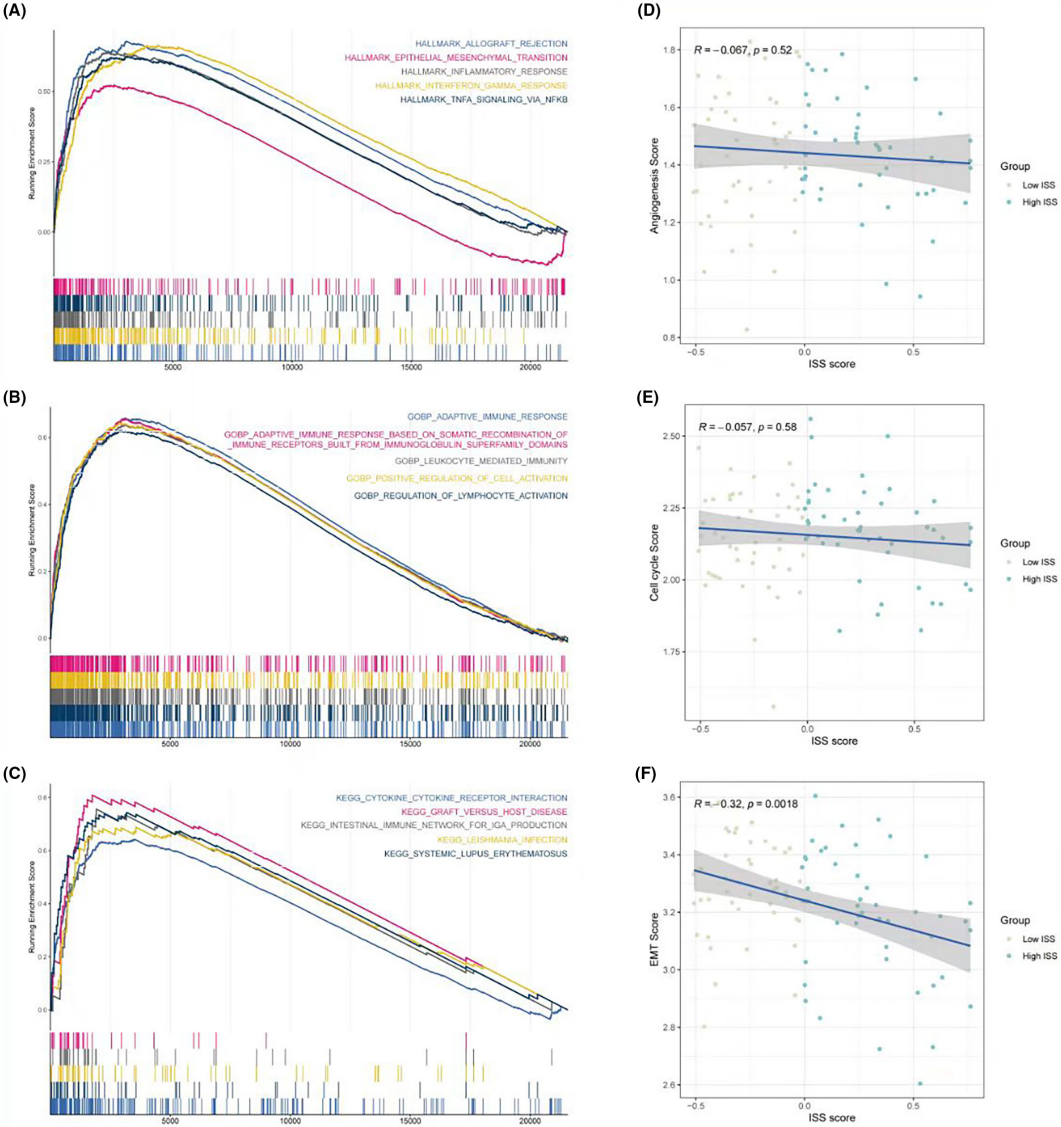
Pathway assessment in relation to ISS scores. (A) GSEA results using the HALLMARK gene set, revealing significant enrichment of EMT and interferon‐related processes in the low ISS score group. (B) GSEA results from the GOBP analysis, indicating significant enrichment of adaptive immune response and lymphocyte activation regulation pathways in the low ISS score group. (C) GSEA results for KEGG pathways, demonstrating significant enrichment of cytokine–cytokine receptor interaction processes in the low ISS score group, consistent with the high immune activity identified in this group. (D–F) Series of plots analysing the correlation between ISS scores and classic tumour‐related processes such as angiogenesis, cell cycle, and EMT, with a significant negative correlation found only with EMT, suggesting that while the low ISS score group has better immune activity, the EMT process may be driving tumour development in these patients.

In terms of pathway assessment, GSEA results of the HALLMARK gene set showed that the low ISS score group significantly exhibited functions like EMT (epithelial–mesenchymal transition) and interferon‐related processes (Figure 6A). GSEA results of Gene Ontology Biological Process (GOBP) indicated that the low ISS score group had significant adaptive immune responses and lymphocyte activation regulation (Figure 6B). Furthermore, GSEA results for Kyoto Encyclopedia of Genes and Genomes (KEGG) demonstrated significant cytokine–cytokine receptor interaction processes in the low ISS score group (Figure 6C), aligning with the previously mentioned high immune activity conclusions of the low ISS score group. Correlation analysis of ISS scores with three classic tumour‐related processes—angiogenesis, cell cycle, and EMT—revealed that only EMT was significantly negatively correlated with ISS scores. This suggests that despite the low ISS score group having better immune activity and responses, the processes driving tumour development might be related to EMT (Figure 6D–F).

### 3.7 Pan‐cancer analysis based on key prognostic genes CD36 and MYD88

**FIGURE 7:**
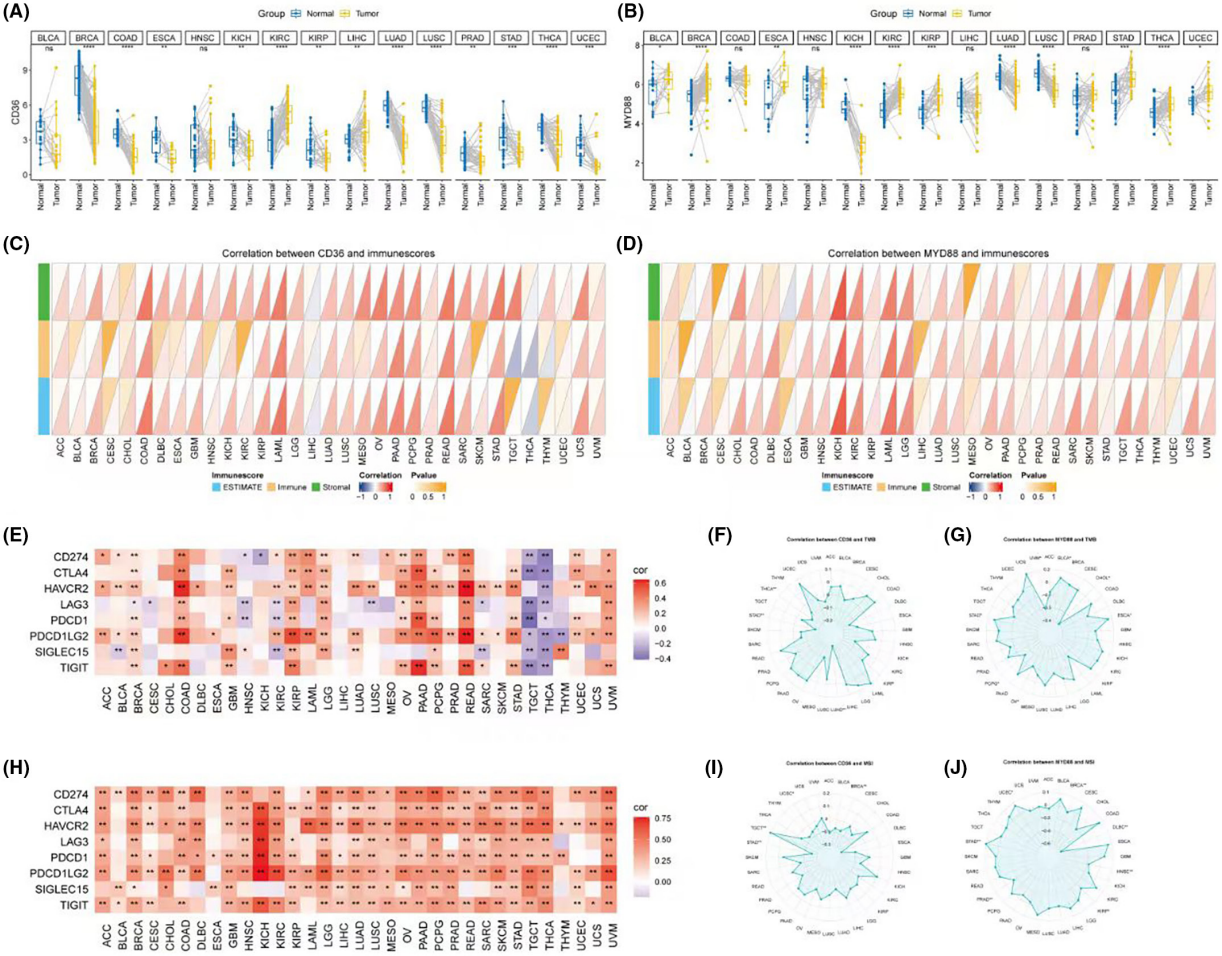
Expression profiles and clinical correlations of CD36 and MYD88 in various cancers. (A) Violin plots comparing the expression levels of CD36 in various cancer types to corresponding control groups, highlighting the higher expression in BRCA, COAD, KIRC and LIHC. (B) Violin plots illustrating the expression levels of MYD88 across different cancer types, with notable upregulation in cancers, such as BRCA and ESCA. (C) Correlation graphs depicting the relationship between CD36 expression and immune, stromal and overall microenvironment scores across various cancers. (D) Correlation graphs showing the association between MYD88 expression and immune, stromal and overall microenvironment scores in different cancers. (E) Heatmap representing the correlation between CD36 expression and the expression of immune checkpoint molecules in various cancers, with significant correlations noted in COAD, PAAD, READ, TGCT and THCA. (F) Heatmap displaying the correlation between MYD88 expression and immune checkpoint molecules across all studied cancers, with a strong positive correlation particularly in KICH. (G) Radar charts depicting the correlation between CD36 expression and tumour mutational burden (TMB) in selected cancers, with significant correlations observed in LUAD, THCA and STAD. (H) Radar charts showing the correlation between MYD88 expression and TMB across various cancers, highlighting significant correlations in BLLCA, ESCA, STAD, CHOL, OV and UVM. (I) Correlation plot for CD36 and microsatellite instability (MSI) across several cancer types, indicating both positive and negative associations. (J) Correlation plot for MYD88 and MSI, with significant positive correlations in STAD and UCEC, and negative correlations in BRCA, DLBC, HNSC, KIRP and PRAD.

Subsequent analysis focused on pan‐cancer studies of the two core prognostic genes, CD36 and MYD88. In terms of expression, CD36 was found to be highly expressed in control groups of various cancers, including BRCA and COAD, but was only highly expressed in KIRC and LIHC (Figure 7A). On the other hand, MYD88 showed upregulated expression in several types of cancer, mainly including BRCA and ESCA (Figure 7B). Regarding the microenvironment, both CD36 and MYD88 showed a significant positive correlation with stromal score, immune score and overall microenvironment score in various cancers (Figure 7C,D). Further evaluating their relevance to immunotherapy, in terms of immune checkpoints, CD36 showed a significant positive correlation in COAD, PAAD and READ, but a significant negative correlation in TGCT and THCA (Figure 7E). MYD88, however, showed a significant positive correlation with checkpoint expression in all cancers, especially in KICH (Figure 7F). Regarding tumour mutational burden (TMB), CD36 was significantly positively correlated only in LUAD, while showing a significant negative correlation in THCA and STAD (Figure 7G); MYD88 was significantly positively correlated with TMB in BLLCA, ESCA, STAD, but the opposite was found in CHOL, OV and UVM (Figure 7H). In terms of microsatellite instability (MSI), it was negatively correlated with CD36 in BRCA, STAD, UCEC and positively correlated in TGCT (Figure 7I); while it was significantly positively correlated with MYD88 in STAD and UCEC, and negatively in BRCA, DLBC, HNSC, KIRP and PRAD (Figure 7J).

**FIGURE 8:**
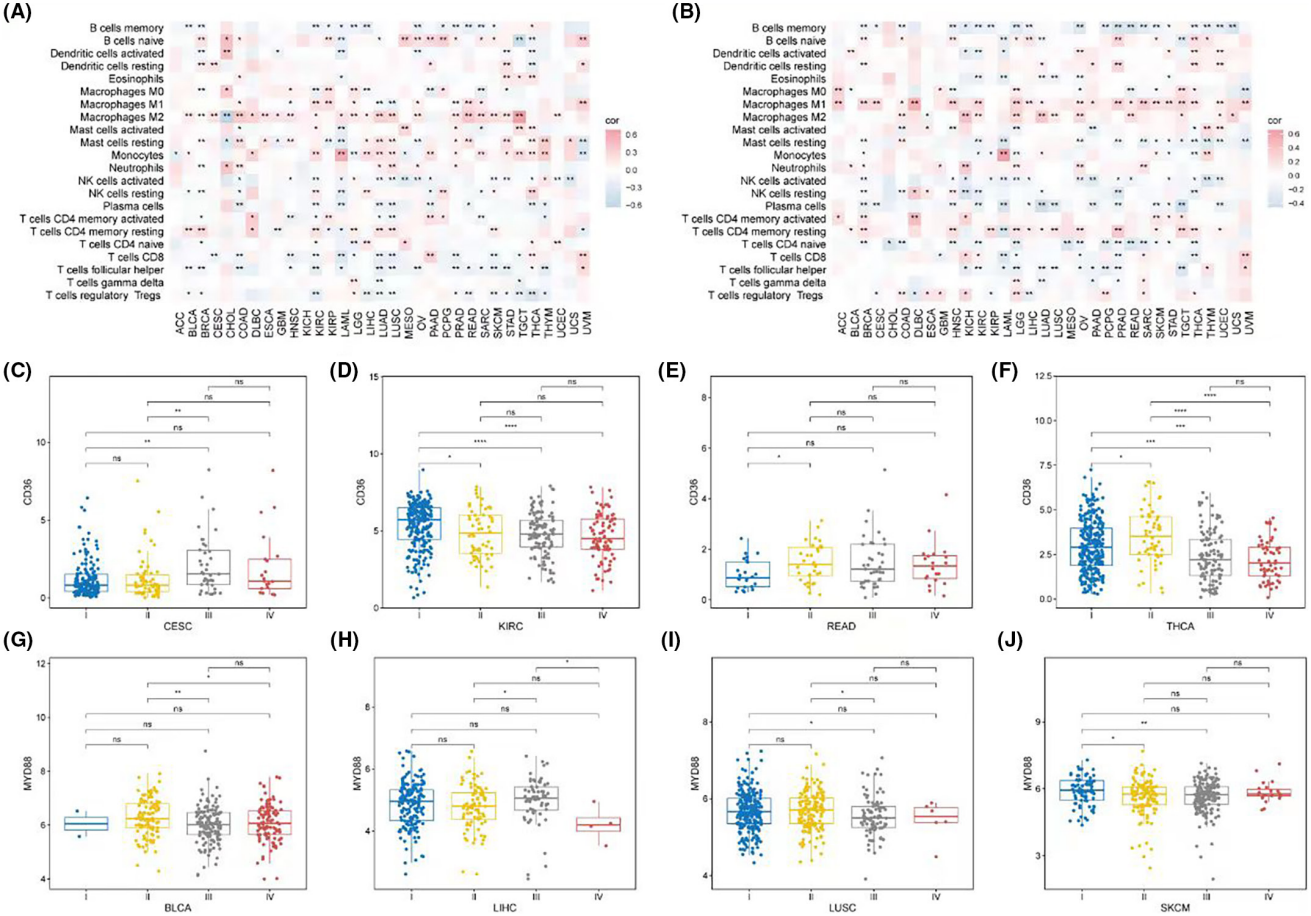
Impact of CD36 and MYD88 expression on immune cell abundance and clinical staging in cancer. (A) Heatmaps reflecting the correlation between CD36 expression and the abundance of various immune cell types, using Cibersort analysis across multiple cancers. (B) Heatmaps showing the correlation between MYD88 expression and immune cell abundances, revealing positive correlations with M1 macrophages and negative correlations with memory B cells, naïve CD4 T cells, and T helper cells. (C–F) Box plots delineating the expression levels of CD36 across different clinical stages in CESC (C), KIRC (D), READ (E) and THCA (F). (G–J) Box plots illustrating the differential expression of MYD88 across clinical stages in BLCA (G), LIHC (H), LUSC (I), and SKCM (J).

In terms of immune cells, a pan‐cancer assessment of immune cell abundance was conducted using Cibersort. CD36 was predominantly positively correlated with M2 macrophage abundance and significantly negatively correlated with memory B cells, CD8 T cells, and T helper cells among most cancers (Figure 8A). MYD88, on the other hand, was primarily positively correlated with M1 macrophages and significantly negatively correlated with memory B cells, naïve CD4 T cells, and T helper cells (Figure 8B). In terms of clinical classification, the expression of CD36 showed differences in stages in CESC (Figure 8C), KIRC (Figure 8D), READ (Figure 8E) and THCA (Figure 8F); while MYD88 showed stage‐based expression differences in BLCA (Figure 8G), LIHC (Figure 8H), LUSC (Figure 8I), and SKCM (Figure 8J).

**FIGURE 9:**
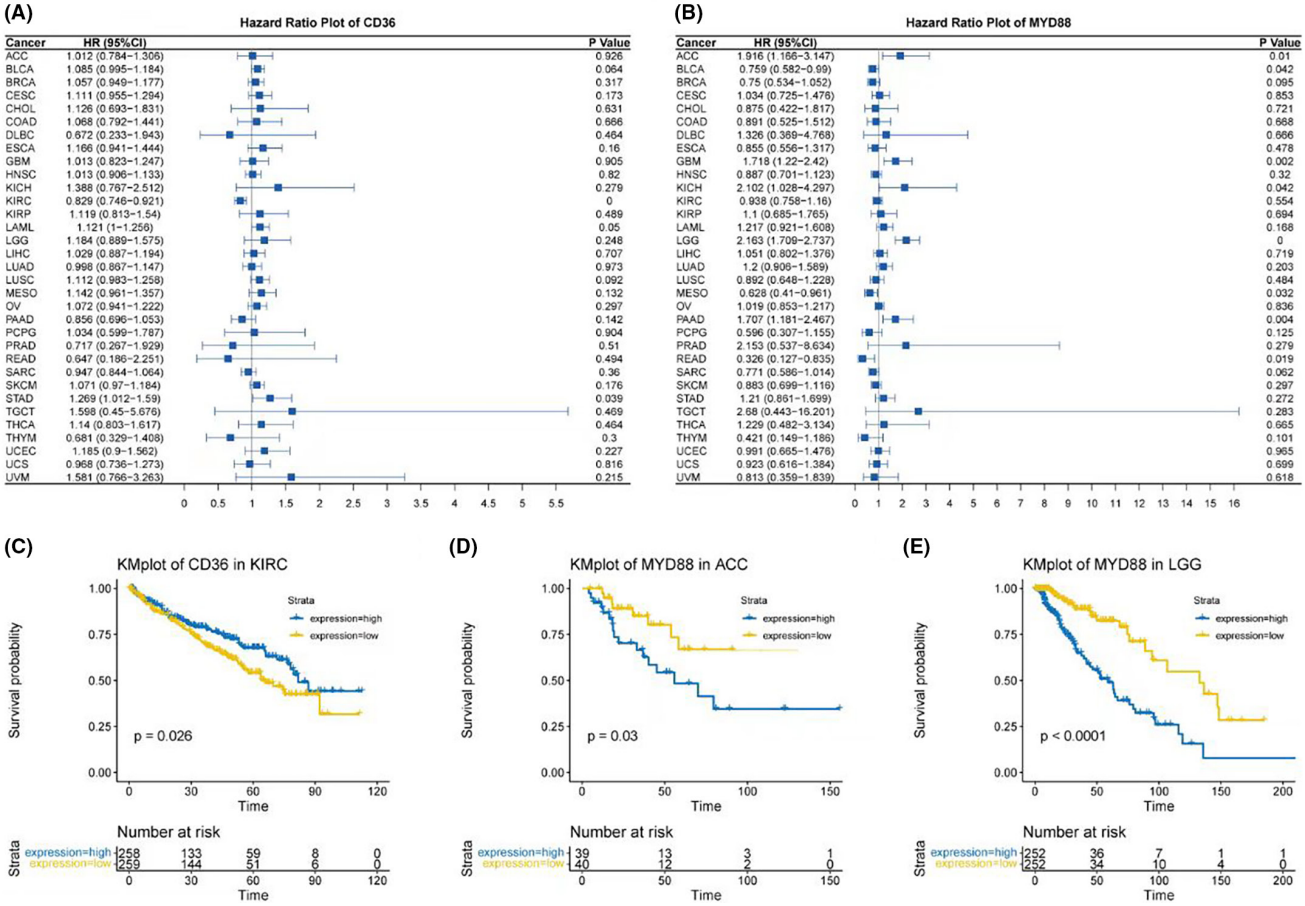
Prognostic significance of CD36 and MYD88 expression across cancer subtypes. (A) Forest plot summarizing the hazard ratios (HRs) for CD36 and its association with survival across various cancer types, with significant relevance observed in KIRC and STAD. (B) Forest plot depicting the HRs for MYD88, highlighting its relationship with survival in several cancers, including ACC, BLCA, GBM, KICH, LGG, MESO, PAAD and READ. (C) Kaplan–Meier survival plot for CD36 in KIRC, stratifying patients into high and low expression groups based on median expression values and showing distinct survival outcomes. (D) Kaplan–Meier survival plot for MYD88 in ACC, demonstrating survival differences between high and low expression groups. (E) Kaplan–Meier survival plot for MYD88 in LGG, also indicating significant survival differences based on expression levels.

Finally, survival analyses were conducted. Forest plots indicated that CD36 had significant survival relevance in KIRC and STAD (Figure 9A), while MYD88 was related to survival in ACC, BLCA, GBM, KICH, LGG, MESO, PAAD and READ (Figure 9B). Stratified survival curves based on the expression of CD36 and MYD88 were plotted for these tumours. In our study, a differential analysis revealed that only in KIRC for CD36, and in ACC and LGG for MYD88, could we significantly stratify patients into high and low expression groups based on median expression levels, which corresponded to distinct survival outcomes. This was demonstrated in Kaplan–Meier plots (Figure 9C–E).

### 3.8 CD36 and MYD88 promote tumour proliferation, invasion and migration in OS

**FIGURE 10:**
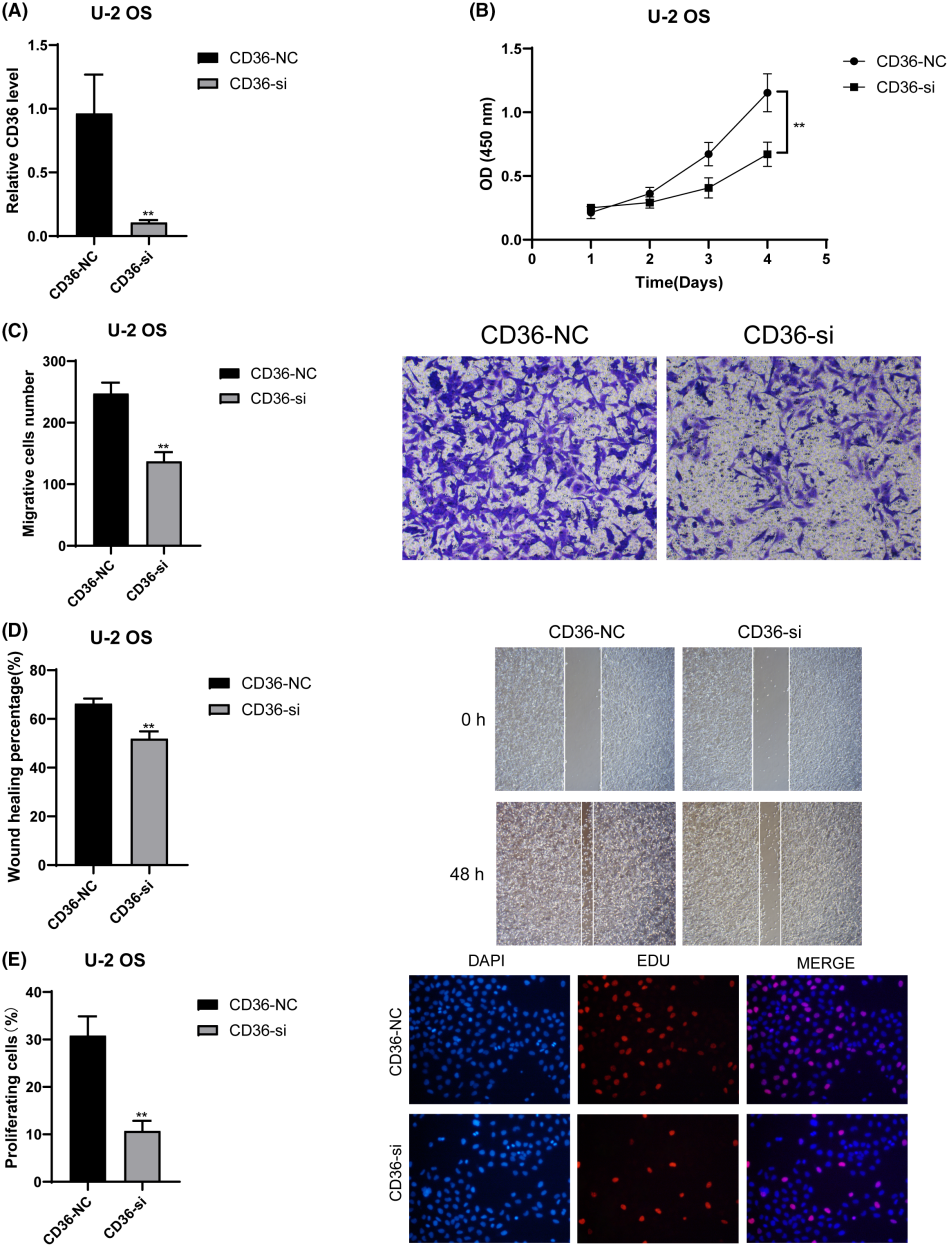
Effects of CD36 knockdown on osteosarcoma cells. (A) RT‐qPCR analysis showing decreased mRNA levels of CD36 in siRNA‐treated cells compared to control. (B) CCK8 assay results indicating reduced cell viability in CD36 siRNA‐treated cells. (C) Transwell assay results demonstrating decreased invasive capability in CD36‐knockdown cells. (D) Wound healing assay results revealing impaired migratory ability of cells with CD36 knockdown. (E) EDU assay results showing reduced proliferation in CD36‐silenced cells. All experiments were conducted in triplicate, and data are presented as mean ± SD. **p < 0.01 indicates significant difference compared to control.

**FIGURE 11:**
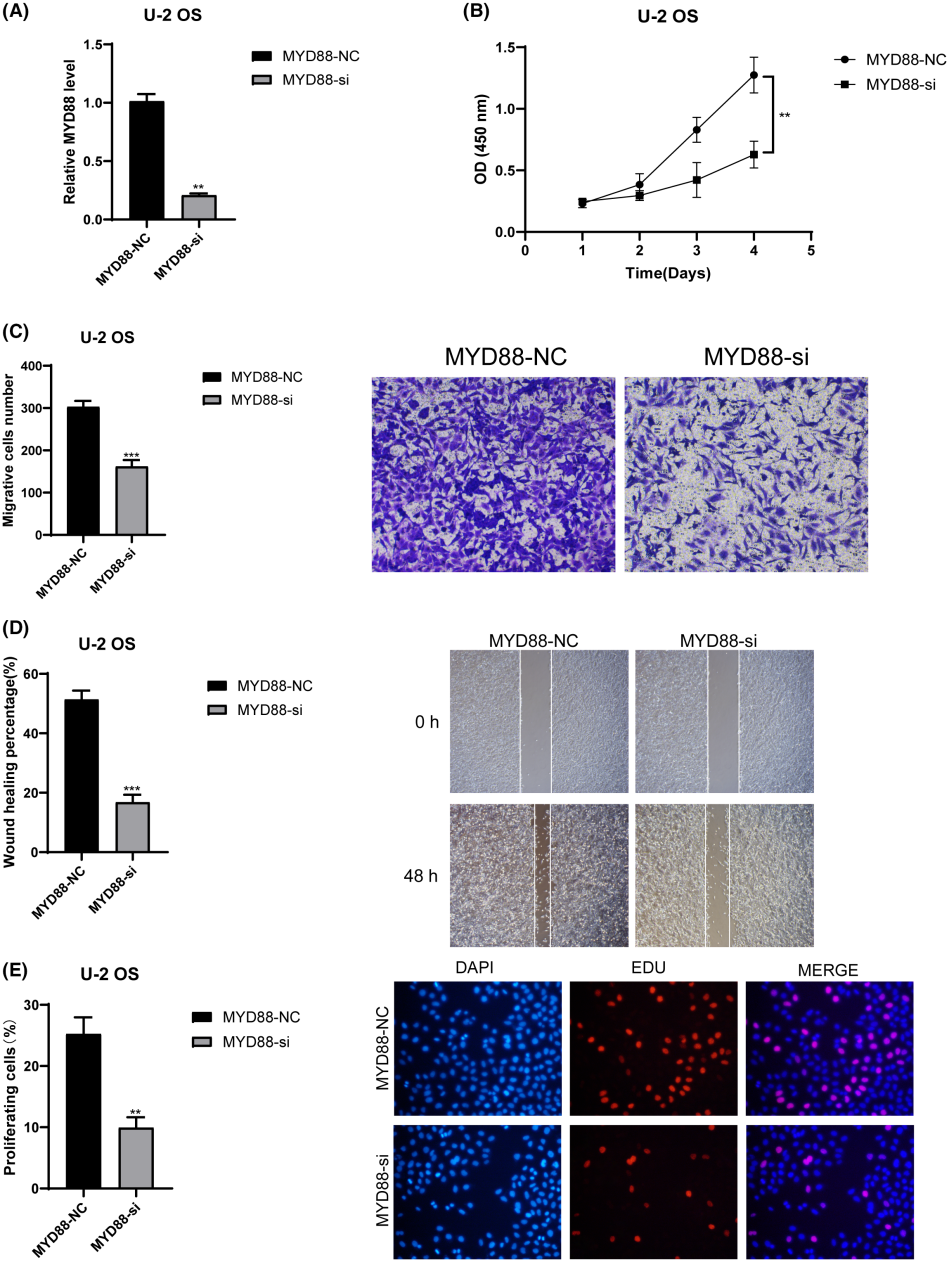
Effects of MYD88 knockdown on osteosarcoma cells. (A) RT‐qPCR analysis displaying decreased mRNA levels of MYD88 in siRNA‐treated cells compared to control. (B) CCK8 assay results showing diminished cell viability in MYD88 siRNA‐treated cells. (C) Transwell assay results illustrating reduced invasive capability in MYD88‐knockdown cells. (D) Wound healing assay results showing decreased migratory ability of cells with MYD88 knockdown. (E) EDU assay results indicating decreased proliferation in MYD88‐silenced cells. All experiments were performed in triplicate, and data are presented as mean ± SD. **p < 0.01 denotes significant difference compared to control.

We validated the roles of CD36 and MYD88 in OS through in vitro experiments. Initially, we utilised siRNA to knock down these two genes individually, and RT‐qPCR results demonstrated a significant decrease in mRNA relative expression levels in the siRNA groups compared to the control groups (*p* < 0.01) (Figure 10A for CD36 and 11A for MYD88). This indicates the high knockdown efficiency of our siRNAs, enhancing the credibility of subsequent studies. Results from CCK8 experiments revealed a significant decrease in cell absorbance after knocking down CD36 (Figure 10B) and MYD88 (Figure 11B) compared to the control groups (*p* < 0.01). Results from Transwell experiments demonstrated a significant decrease in the invasive ability of OS cells after knocking down CD36 (Figure 10C) and MYD88 (Figure 11C) (*p* < 0.01). Wound healing experiments indicated a significant decrease in the migratory ability of OS cells after knocking down CD36 (Figure 10D) and MYD88 (Figure 11D) (*p* < 0.01). Similarly, in the EDU experiment, fluorescence intensity significantly decreased after knocking down CD36 (Figure 10E) and MYD88 (Figure 11E) (*p* < 0.01). These findings collectively indicate that knocking down CD36 and MYD88 significantly reduces the proliferative activity of OS cells, thereby confirming the role of CD36 and MYD88 in promoting OS proliferation. In conclusion, through wet experiments validation, we demonstrate that CD36 and MYD88 promote tumour proliferation, invasion, and migration in OS.

## 4 DISCUSSION

In our study, we meticulously explored the complex interplay between inflammasome‐related genes and the prognosis of osteosarcoma, employing an innovative approach by integrating multiple machine learning techniques to refine our predictive model. The application of the RSF model emerged as a potent predictor, yet the disparity between the training and validation sets' c‐index values urged us to adopt a more stable combination, leading to the utilisation of StepCox for variable selection and GBM for model construction. This methodological pivot highlighted CD36 and MYD88 as pivotal genes, underscoring their significant influence on the ISS score and, by extension, on overall survival rates.

The association between specific inflammasome gene signatures and poor patient outcomes observed in our study aligns with the growing body of research that identifies inflammation as a key player in cancer. This relationship is now increasingly recognised as a factor that contributes not only to the initiation of cancer but also to its progression and metastasis.^37^, ^38^ This dual functionality of inflammation—both as a defence mechanism and a facilitator of tumour growth—highlights the complex interplay within the tumour microenvironment and signifies a need for a nuanced approach to cancer therapy.

In contrast to some studies that did not find a significant correlation between inflammasome‐related genes and patient survival, our study's robust statistical framework and comprehensive genetic profiling have yielded insights that substantiate the prognostic relevance of these genes in osteosarcoma.^39^ The discrepancy between our findings and those of more limited analyses accentuates the importance of expansive genetic screening in capturing the true impact of these genes on disease outcomes.

Expanding on previous research, our study broadens the genetic spectrum associated with osteosarcoma by uncovering additional inflammasome‐related genes. This comprehensive identification enhances our understanding of osteosarcoma's complex genetic framework, providing insights into previously underexplored areas of its pathogenesis. The broader genetic perspective uncovered in this study not only enriches our knowledge base, but also paves the way for novel therapeutic strategies that might have been overlooked in narrower investigations.

Furthermore, the prognostic significance of inflammasome‐related genes extends beyond mere risk assessment to encompass their potential as predictive biomarkers for treatment responses. Our findings suggest the exciting prospect of customising osteosarcoma therapies based on distinct inflammasome gene signatures. This approach aligns with the emerging paradigm of precision medicine, which has shown promise in enhancing treatment efficacy and reducing adverse effects in various cancers. The integration of genetic profiling into routine clinical practice could revolutionise the management of osteosarcoma.

Our examination of CD36 and MYD88 as therapeutic targets reveals a compelling avenue for personalised tumour therapy. Specifically, their roles in the regulation of the inflammasome within the osteosarcoma microenvironment warrant attention. CD36, known for its involvement in fatty acid metabolism and angiogenesis, has been implicated in inflammasome activation, which is pivotal in osteosarcoma's inflammatory and metastatic processes.^40^ Similarly, MYD88, serving as a central adaptor protein in TLR and IL‐1 receptor pathways, has a significant impact on inflammasome activity.^41^ It influences the maturation and secretion of inflammatory cytokines, which are crucial in osteosarcoma pathogenesis. The ability of these molecules to modulate the tumour microenvironment suggests they may affect tumour growth, metastasis, and immune escape mechanisms. The rationale for focusing on CD36 and MYD88 as therapeutic targets is underpinned by studies that demonstrate their involvement in tumorigenic processes and their specific impact on inflammasome regulation, which can significantly influence the progression and aggression of osteosarcoma. For instance, evidence suggests that CD36 may contribute to the metastatic process in several cancers,^42^ while MYD88 mutations have been implicated in the dysregulation of immune responses in cancer pathogenesis.^43^ Despite the theoretical promise of these targets, empirical evidence is essential to transition these strategies from bench to bedside effectively, warranting further research to validate these approaches in a clinical context.

This study, while providing valuable insights into the role of inflammasome‐related genes in osteosarcoma, has its limitations. First, the reliance on retrospective data may introduce inherent biases that could affect the generalizability of the findings. Additionally, the study's conclusions are based on computational analyses and bioinformatics predictions, which, although informative, require further validation through experimental studies to confirm the functional roles of the identified genes and their impact on osteosarcoma pathogenesis and treatment response. The sample size, although adequate for exploratory analysis, might not fully capture the heterogeneity of osteosarcoma, potentially limiting the robustness of the findings. Moreover, the study focuses predominantly on genetic factors, overlooking other crucial aspects, such as epigenetic modifications, tumour microenvironment and patient lifestyle factors that could significantly influence disease progression and treatment outcomes. Finally, the translation of these findings into clinical practice necessitates comprehensive clinical trials to assess the safety, efficacy and feasibility of personalised treatments based on inflammasome gene profiles.

In sum, our investigation into inflammasome‐related genes in osteosarcoma not only reinforces their proposed significance in cancer research but also challenges some established notions by presenting a comprehensive view of their prognostic capabilities. The potential therapeutic implications of our findings are substantial, inviting a re‐evaluation of current treatment paradigms and suggesting a more active role for these genes in the narrative of cancer progression. The road ahead is one of cautious optimism, as we look towards a future where our expanding knowledge of osteosarcoma genetics translates into real‐world benefits for those afflicted by this disease.

## AUTHOR CONTRIBUTIONS

**Nan Zhang:** Data curation (equal); formal analysis (equal); writing – original draft (lead); writing – review and editing (supporting). **Ying Han:** Data curation (equal); formal analysis (equal); writing – review and editing (supporting). **Hangkai Cao:** Data curation (equal); formal analysis (equal); writing – review and editing (supporting). **Qingxin Wang:** Conceptualization (lead); writing – review and editing (lead).

## FUNDING INFORMATION

The article was funded by Jiaxing People's Livelihood Science and Technology Innovation Research Project Fund (SQGY202300254) and Science and Technology Bureau of Jiaxing City (2023AY31017).

## CONFLICT OF INTEREST STATEMENT

The authors declare that the research was conducted in the absence of any commercial or financial relationships that could be construed as a potential conflict of interest.

## Data Availability

The original contributions presented in the study are included in the article/supplementary material, further inquiries can be directed to the corresponding author.
